# Globalisation and neoliberalism as structural drivers of health inequities

**DOI:** 10.1186/s12961-018-0365-2

**Published:** 2018-10-09

**Authors:** Rama V. Baru, Malu Mohan

**Affiliations:** 1 Centre of Social Medicine and Community Health, Jawaharlal Nehru University, New Delhi, India; 2 Achutha Menon Centre for Health Science Studies, Sree Chitra Tirunal Institute of Medical Science and Technology, Trivandrum, India

**Keywords:** Neoliberalism, Philanthrocapitalism, Globalisation, Health inequities in India, Health equity research, Structural drivers, AMCCON 2018

## Abstract

In this paper, we draw upon and build on three presentations which were part of the plenary session on ‘Structural Drivers of Health Inequities’ at the National Conference on Health Inequities in India: Transformative Research for Action, organised by the Achutha Menon Centre for Health Science Studies in Trivandrum, India. The three presentations discussed the influential role played by globalisation and neoliberalism in shaping economic, social and political relationships across developed and developing countries. The paper further argues that the twin process of globalisation and liberalisation have been important drivers of health inequities. The first segment of the paper attempts a broader conceptualisation of neoliberalism beyond the economic realm. Using Stephanie Lee Mudge’s conceptualisation (*Soc Econ Rev* 6:703–3, 2008) we have analysed how the political, bureaucratic and intellectual domains of neoliberalism have intersected and redefined the role of state and commercialised health services leading to inequities. Neoliberal ideas have reconfigured the role and changed the priorities of non-governmental organisations resulting in a fracture within this movement. n the second segment, we focus on the rise of American philanthro-capitalism, and how the two major foundations, the Rockefeller Foundation (early twentieth century) and the Bill and Melinda Gates Foundation (twenty-first century), have shaped the ideology of institutions engaged in international health and influenced the global health agenda. We discuss how the activities of philanthro-capitalists have transformed the architecture of health governance through their top-down organisational culture and deficit of structures to ensure accountability. The third and final segment of the paper focuses on how neoliberalism as a political project and cultural movement has forged alliances with conservative politics and religious fundamentalisms, resulting in negative consequences for women and other marginalised groups. These alliances have resulted in the control of women’s bodies and contributed to the reversal of hard-won rights for health and gender justice in many parts of the world.

## Background

This paper draws upon and builds on the presentations in the plenary session on ‘Structural Drivers of Health Inequities’ at the National Conference on Health Inequities in India: Transformative Research for Action, organised by the Achutha Menon Centre for Health Science Studies in Trivandrum, Kerala. It elaborates on how globalisation and neoliberal ideas played a dominant role in reconfiguring economic, social, political and institutional relationships that have profoundly impacted people’s lives.

There are many ways in which the term neoliberalism has been used for analysing its influence on the structure of health services. Much of the scholarly and populist writings have conceptualised neoliberalism as a hegemonic ideological project. For a more meaningful analysis, we need to conceptualise neoliberalism in broader terms. It is essential to go beyond the economic aspects and encompass its effects on political, cultural and social processes.

**Fig. 1:**
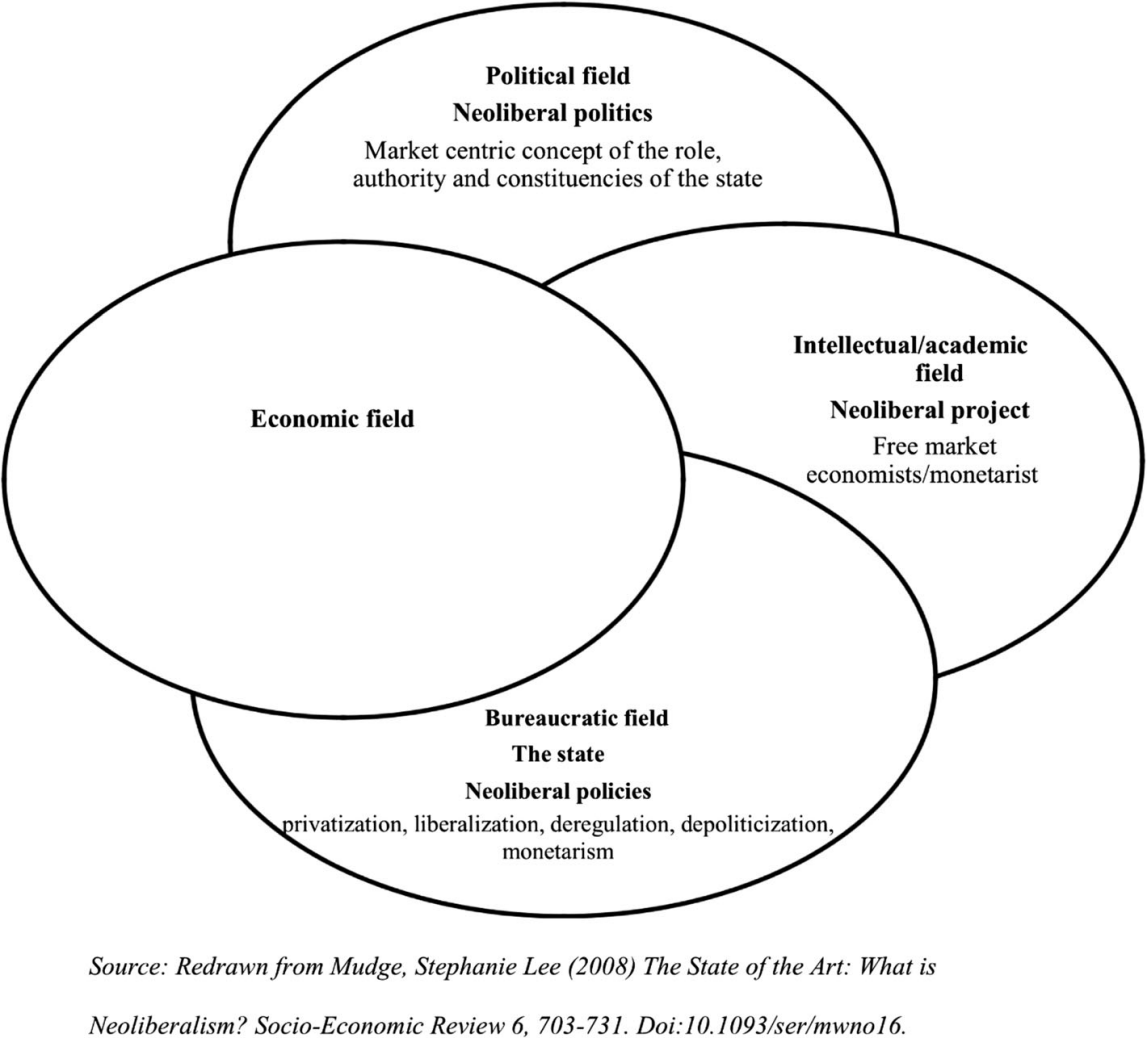
Mudge’s conceptualisation of neoliberalism. Redrawn from Mudge [1]

Stephanie Lee Mudge’s [1] conceptualisation of neoliberalism helps us to delineate the processes through which neoliberalism influences health inequities (Fig. 1). According to Mudge, there are three interconnected faces, namely the political, bureaucratic and intellectual or academic faces. She points out that considering the complex interaction between the three faces is necessary for any analysis of how neoliberalism has impacted the individual, institutions, culture, social and economic relations. Here, she emphasises how neoliberal economic policies are in fact political. As she elaborates:

1. “*Neoliberalism’s intellectual face is distinguished by (a) its Anglo-American anchored trans-nationality; (b) its historical gestation within the institutions of welfare capitalism and the Cold War divide and (c) an unadulterated emphasis on the (disembedded) market as the source and arbiter of human freedoms*”.
2. “*The bureaucratic face is expressed in State policy: liberalisation, deregulation, privatisation, depoliticisation, and monetarism. The family of reforms is targeted at promoting unfettered competition by getting the State out of the businesses of ownership and getting politicians out of the business of dirigiste–style economic management. Neoliberal policies also aim to ‘desacralise’ institutions that have formerly been protected from the forces of private market competition, such as education and healthcare*”.
3. “*Its political face is a new market-centric ‘politics’, i.e. struggles over political authority that shares a particular ideological centre or, in other words, are underpinned by an unquestioned ‘common sense’. On the elite level, neoliberal politics is bound by certain notions about the state’s responsibilities (to unleash market forces wherever possible) and the locus of state authority (to limit the reach of political decision-making). They also tend to be oriented towards specific constituencies (business, finance and white-collar professionals) over others (trade unions, especially)*” [1].

This conceptualisation is useful in describing how the various faces of neoliberalism have intersected and redefined the role of the state and society.

In the analysis of neoliberalism and health, there is a great deal of conceptual confusion. A quick review of Indian studies shows that they pay more attention to neoliberalism as a hegemonic ideological project. Several studies have examined how neoliberal policy instruments, such as privatisation, marketisation, commercialisation and deregulation, have led to the expansion of markets in the economic and social sectors [2, 3]. In the case of the health sector, this has meant the restructuring of the public sector by introducing market principles and reducing the barriers for movements of capital to invest in for-profit services. Several studies have identified the critical role played by global multilateral organisations like the World Bank and the International Monetary Fund (IMF) in furthering neoliberalism through the Structural Adjustment Programmes (SAP) in India [2, 4]. Thus, the introduction of SAPs served as the starting point for critical academic writing on neoliberal reforms in health. An impositional perspective^1^ to the engagement of the World Bank and the Indian state informed most of these studies [2–5]. This perspective argues that the World Bank coerced the Indian government to accept a range of neoliberal conditionalities that were tied to the loans. An impositional perspective is limiting because it gives undue importance to the power wielded by international financial institutions. The Indian government did not passively accept the conditionalities but rather negotiated their terms with the World Bank. Therefore, one would disagree with the view that the World Bank coerced the Indian government, which does not give agency to the Indian state.^2^

It is also important to note that, unlike some countries in Africa, in the Indian case, neoliberal ideas were in circulation, with opposition and resistance to these ideas even before their introduction in 1991 [6].^3^ However, the elite across major political parties and the administration, including the medical and non-medical civil services, academia and civil society, was in sync with the neoliberal ideology and played an active role in supporting the idea and content of SAPs [5, 7].

The shifts in the ideological position of large sections of the elite to support liberalisation broadly correspond to the academic and intellectual field of the face of neoliberalism as conceptualised by Mudge. The intellectual field played an essential role in shaping policy by legitimising liberalisation and privatisation. Several influential academics, policy and media analysts played an active role in furthering these ideas.^4^ It is interesting to note that persons who were advocates of free-market philosophy held several of the critical policy portfolios in finance, industry, education and health. Some of them had held senior positions in the World Bank, IMF and WHO prior to occupying influential positions in government. Thus, there was an epistemic community of Indian academics, civil servants, doctors and scientists, including their diasporic counterparts, who subscribed to the ideology and instruments of neoliberal policy even before the formal introduction of SAPs by the Bretton Wood Institutions.

While one could argue that the year 1991 is a marker for the introduction of structural adjustment policies of the World Bank and IMF, the conditionalities of SAP further accelerated the liberalisation and privatisation of the Indian economy. There were two-forms of loans offered by the World Bank, including hard loans for economic restructuring and soft loans for reforming the social sectors; the former was loaned on interest while the latter was offered with practically zero interest. Both these loans were tied to some conditionalities that encouraged the restructuring of these sectors towards market-friendly policies.

Many of these conditionalities played an important role in shaping health policy from the 1990s onwards. Health sector reforms were introduced into a largely underfunded, weak public sector that co-existed with an aggressively growing private sector. The public sector was reconfigured with the introduction of market principles. Some of the important elements included the introduction of user fees, public–private partnerships and greater decentralisation of the health service system. One of the most visible effects of the neoliberal policies was the commercialisation of health services.

The concept of commercialisation is useful because it allows us to analyse the circulation and accommodation of private capital in the public, for-profit and non-profit sectors [3]. It acknowledges how the accommodation of private capital fundamentally changes the character and culture of public and non-profit institutions. Over time, the non-profit institutions start thinking and behaving more like for-profit institutions. This change in mindset has serious consequences for the availability, accessibility, affordability, acceptability and quality of health services. Over the last two decades, the negative fallout of commercialisation in developed and developing countries has been the widening of inequalities in access. The burden of paying for care has affected the lower middle and working classes adversely. Rising out-of-pocket and catastrophic expenditures on medical care in India is an example of inequities in access. High out-of-pocket expenditure in health results in a significant proportion of the population foregoing medical treatment when it is most needed due to the inability to pay [8].

Health and health service inequities became global concerns a decade after the initial euphoria of neoliberalism. Several countries in Africa, Latin America and Asia that had taken loans under SAPs had implemented health sector reforms and were faced with the challenge of rising inequities in access. Many public health and social movements highlighted these inequities on a global platform.^5^ Even Economists like Joseph Stiglitz, who was an advocate of neoliberalism, wrote on the discontents of globalisation and highlighted the fault lines of liberalisation and globalisation across and within the developing and developed countries [9]. His concerns partially resonated with the social and public health movements across countries that gave voice to those who were excluded from the fruits of globalisation and liberalisation. It is in this context that the concern about development enters the global discourse with the formulation of Millennium Development Goals. Several of these goals directly addressed health issues and many others addressed the social determinants of health. Subsequently, the Asian and global financial crises proved to be a setback to the juggernaut of neoliberal policies. It became increasingly clear that the fruits of globalisation and liberalisation benefitted few, resulting in a significant proportion of the population being ‘left out’ regarding access to markets, employment and basic social security services. This was a concern for those who supported as well as those who opposed liberalisation [6]. The former saw these tendencies as a potential source of destabilisation, while the latter were concerned about the injustice of the neoliberal project.

### Neoliberalism and the reconfiguration of non-governmental organisations (NGOs)

Scholarly writings have largely focused on the growth of the private sector and the restructuring of the public sector during the last three decades. Nevertheless, little attention has been paid to the transformation of non-governmental organisations (NGOs) during this period. It is often assumed that NGOs were homogeneous and unaffected by neoliberal ideas and commercialisation. However, this was not the case. The policies of the World Bank and IMF reconfigured the role of NGOs in the health sector. Greater importance was given to the role of NGOs as facilitators and as representing the voice of the people, as compared to the role and representation of the state. They were given a special place in policy and programme implementation. For example, public–private partnerships became an important element in national disease control programmes like HIV/AIDS, tuberculosis, malaria and leprosy. Kapilashrami and McPake [10], in their critical study of the role of the Global Fund to fight Tuberculosis, AIDS, and Malaria in five states of India, observed that the funding made available through these global initiatives created many distortions and fissures within the NGO community. It led to unhealthy competition in getting access to resources.

The increased fund flow influenced and changed the priorities of several NGOs. Global agendas started shaping the priorities and activities of NGOs at the national and local levels. This resulted in a fracture within the NGO movement with a significant number of them indulging in doublespeak. Radical rhetoric and the language of rights was a façade for legitimising neoliberal policies. There was a strong move to delegitimise the role of the state, which proved to be beneficial for the growth of the for-profit sector. As Utting observes [11], many of the NGOs were furthering the agenda of commercialisation through their activities and advocacy. This was an important development in the transformation of the NGO sector in health. The earlier understanding was that NGOs played a major role in resisting the neoliberal agenda. However, over a period, NGOs became an ‘ideologically fractured landscape’ [12].

### The new avatar of external funding in health: the rise of American philanthro-capitalism

The role of the World Bank and IMF in the health sector lost its sheen during the last two decades. The global and Asian financial crises challenged World Bank policies, leading to the decline in the power and influence that was once wielded by the Bretton Wood institutions. In the health sector, the adverse effects of reforms as prescribed by the World Bank were being seen across developing countries [13]. There was evidence to show that the commercialisation of public health services led to inequities in access. Various progressive social and health movements drew attention to the ill effects of commercialisation across the globe. Several of these movements formed global alliances and forged campaigns for resisting World Bank policies.^6^ The ongoing criticism and coordinated action did not come from the radical movements alone but found a voice from the advocates of globalisation and liberalisation who were concerned about growing socioeconomic inequities. The United States, which was a major contributor to the multilateral organisations, including WHO, started reducing its share due to the growing resistance to SAPs. As a result, the UN and WHO were faced with a financial crisis. At this point, private capital in the form of pharmaceutical industry and philanthro-capitalist groups like the Bill and Melinda Gates Foundation (BMGF) entered global health. Global public–private partnerships were forged for several disease control programmes and for the production of vaccines. The autonomy and normative role of WHO was compromised by the entry of big capital [14].

American foundations have historically played an important role in international health. The earliest among them is the Rockefeller Foundation, of the early twentieth century. Birn traced the history and transformation of the American philanthropists and their engagement with health [15]. She reminds us that, while philanthro-capitalism has been defined as a desire to commit for the welfare of the others or to invest money to good causes, it does not call for loving all humans sincerely equally or loving them more than making money. She clarifies this concept, by citing a comment made by Rockefeller Junior that he was not in the business of producing oil, but was in the business of making money. The fundamental objective of corporations is to maximise benefits and profits for their shareholders. Birn’s research writings on this subject document how two major American foundations, namely, the Rockefeller Foundation, of the early twentieth century, and the BMGF, of the twenty-first century, have shaped the ideology of institutions in practices of international and global health. These two entrepreneurs and foundations shared a belief in narrow technology-centred biomedical approaches and tended to overlook the social, political and economic determinants of health. Both of them extended their medical empire into education, agricultural and natural sciences and development.

However, a critical difference that divides them is that the Rockefeller Foundation historically supported government responsibility in public health. The Rockefeller Foundation also favoured the creation of a single multilateral coordinating agency for global health which later took shape as the WHO. The BMGF is the world’s largest philanthropic organisation wit the ability, through its diverse business interests, to mobilise funds that many multilateral organisations may not be able to. As Birn observes [14], the BMGF made endowments to the extent of 40 billion dollars in 2016, exceeding the 20 billion dollars so far donated by the United States mega-investor Warren Buffet. The United States public underwrites at least a third of this endowment with no say in its policies. The BMGF spends more on global health than any governments except the United States, with its spending in some years exceeding that of WHO. In fact, WHO receives funding from BMGF and has had to restructure several of its disease control and vaccine development programmes into Global Public–Private Partnerships. Further, the BMGF played an important role in the formation of the H8, which is similar to the G8. The H8 consists of WHO, UNICEF, UNFPA, UNAIDS, the World Bank, the BMGF, the GAVI Alliance, and the Global Fund to Fight AIDS, Tuberculosis, and Malaria. Birn further points out, that: “*The H8 holds meetings, like the G8, at which the mainstream global health agenda is shaped behind closed doors, and organisations considerably influenced by Gates and the BMGF constitute a plurality*” [15]. Researchers have mapped the institutions and individuals who have received funding from the BMGF and shown that involves partnerships with pharmaceutical, medical device and IT industries [16]. The large volume of funding gives the BMGF power over priority setting for research and policy in health. In recent years, there have been several examples of the BMGF influencing the Indian government to introduce vaccines for childhood immunisation, injectable contraceptives and the like. Citing the experience of the engagement of the BMGF with the state government of Bihar, Birn argues that there is very little interaction between the BMGF, the health system, and its users [16]; the culture of the organisation is top-down with little scope for dialogue. Conflicts of interests have also been pointed out within the BMGF and in many other philanthro-capitalists for investing in polluting industries or unhealthy food and beverages, which are detrimental to public health.

The entry of philanthro-capitalists has transformed the architecture for global health governance and created deficits in the structures for accountability. There is an asymmetry of power within the global order. A great number of large corporations and the wealthiest people in the world, including Gates, Buffet, Bloomberg, Ellison, Zuckerberg, Bezos and Slim, are involved in global health at some level. None of these players are accountable to their shareholders, individual nations or citizens and there are no structures to scrutinise their actions.

The political face of neoliberalism has fundamentally transformed the relationship between big capital and the nation-state, as well as that between the nation-state and its citizens. In this process of reconfiguration and transformation, unholy alliances are forged between business interests and conservative politics, including religious fundamentalism. Therefore, what we wish to illustrate in the next section is that the engagement of neoliberalism with the political face has negative consequences for women and marginalised groups. These consequences result in the control of women’s bodies and jeopardise their sexual and reproductive health and rights.

## Beyond health services: neoliberal globalisation as a driver of gender-based health inequities through its partnership with religious fundamentalisms

In this section, we discuss the pathways through which neoliberal globalisation has influenced the political field. We focus on the rise and resurgence of religious fundamentalisms as a structural driver of gender-based health inequities [17].

### Neoliberalism as a cultural movement and political project

Mudge’s framework helps to extend the analysis beyond the economic dimension of neoliberalism [1]. The rise or resurgence of religious fundamentalisms is a political project of neoliberalism that is influenced by the discontents of liberalisation and globalisation. Evidence from many countries across the globe suggests that liberalisation has benefitted the middle and upper-middle classes disproportionately [18], whilst a substantial section of the population has been left out or dispossessed. It is well acknowledged that this results in rising aspirations and anxieties when aspirations are not fulfilled. Neoliberal globalisation has also contributed to redefining social relationships at the level of the individual, family, community and society. The idea of social solidarity is increasingly being replaced by individualised solutions. As Giroux observes [19], the concepts of social justice, redistribution and democratic citizenship are trumped by consumerism, market efficiency and individual-driven rigorous competition. Consumption becomes the mark of citizenship, and all those individuals and communities who are incapable of consumption and competition become ‘disposable’ [20]. The delineation and the increasing emphasis placed on the ‘private’ over the ‘public’ and the ‘personal’ over the ‘political,’ is one of the defining traits of the neoliberal discourse. Exclusion of sections of the population like women and sexual, gender and religious minorities from civic participation is a characteristic [20, 21] and contributes to marginalising their rights in the personal and political spheres [22].

### Pathways to the rise of religious fundamentalisms

The use of the term ‘religious fundamentalism’ has been widely debated on several grounds. Several arguments like the difficulty in arriving at a shared definition, the reinforcement of ‘negative stereotypes’ and the targeting of Muslims, in particular, have been raised against the use of the term. Religious fundamentalism is transnational, and it shares commonalities across contexts. However, it is also greatly influenced by culture, ethnicity and other local and national factors. Hence, many scholars prefer the use of the term ‘religious fundamentalisms’ (in plural) rather than ‘fundamentalism’ (in singular) to reflect its plurality and context, unless one is referring to the generic concept of fundamentalism, which applies to many religious and non-religious phenomena. For this paper, we define religious fundamentalisms as concerted efforts to bring ‘religion’ as a determinant of policy-making and governance. Bringing religion into policy-making is achieved through strategic manipulation of religion by state and non-state actors for power and control over rights [23].

Capitalist exploitation and a market-driven economy often contributed to increasing poverty, rising inequalities, alienation, loss of identity, violence, economic insecurities and massive dislocations. The opposition that rose from the majority challenged the legitimacy of governments and often governments responded by restricting the democratic space or by counter mobilisation to promote a new sense of solidarity using religion, nationalism and fear of the other (migrants).

### Neoliberal globalisation and religious fundamentalisms as structural drivers of gender-based health inequities

A key area where religious fundamentalisms operate through state policies to contribute to gender-based health inequities is women’s sexual and reproductive health. One of the most widespread consequences of religious interference to gender-based health inequities is the avoidable mortality and morbidity from unsafe abortion despite the availability of medical technology for safe termination of pregnancy. In many parts of the world, abortion services are restricted and criminalised on religious grounds, forcing women to avail unsafe and illegal abortions, thereby endangering their life and health. The discourse around access to abortion services prioritises the religious understanding of what the beginning of life is over the life and choices of women [17, 23].

The criminalisation of homosexuality is another example of how religious fundamentalist positions influence state policy to violate the right to privacy of individuals. People with different sexual orientation are also at the receiving end of negative stereotyping, stigmatising and violence by the social, religious and political structures that are aligned with one another, impacting on their health and wellbeing.

Additionally, access to services may be restricted due to religious groups taking on the role of gatekeepers of women’s morality. For example, in several countries, family planning service providers require husbands’ consent for providing services to women, suggesting that married women are viewed as the possessions of their husbands. Lack of access to sexual and reproductive health services for the young and unmarried arises from the prevailing view that the purpose of sex is primarily for procreative reasons within the institution of marriage and sex outside of marriage is considered immoral.

In the Philippines, because of the opposition from Catholic hierarchy and pro-life groups, it took almost 14 years for the Responsible Parenthood and Reproductive Health Act (2012) to be passed. The Act guarantees universal access to methods of contraception, fertility control, sexuality education and maternal care. Religious groups created barriers asking the Department of Health to go through a judicial process of certifying every single contraceptive as not an abortifacient. The implementation also continues to be plagued by litigations from these same groups [23].

Thus, gender justice and sexual and reproductive health and rights are seriously hampered by religious fundamentalisms, reversing the hard-won rights fought by women’s movements to assert control over their bodies.

## Conclusions

This paper has tried to unpack the influence of neoliberalism and the pathways that have influenced rising inequities in health and access to healthcare. We employed Mudge’s framework to delineate how the three faces of neoliberalism have interacted to reconfigure global, national and local policies. We have demonstrated how health sector reforms were an offshoot of the economic reforms in India. While acknowledging the important role of the World Bank and IMF in furthering the policies for liberalisation, the paper rejects the impositional argument. A more nuanced understanding of the class character of the state, politics and the role of the bureaucratic and academic elites helps explain the endorsement and uptake of neoliberal ideas even before the introduction of SAPs. The present study uses the concept of commercialisation to analyse the process of restructuring of the public, for-profit and non-profit sectors in health services, and argues that commercialisation was an important driver of inequities in the availability, accessibility, affordability and acceptability of health services. While there has been some acknowledgement of the negative fallout of liberal health sector reforms, there is no sign of reversal. Instead, there is an attempt to fix inequities through weakly targeted interventions for the poor that ignore the presence of a social gradient in inequalities in health outcomes and access to health services.

At the global level, there is a shift in patterns of funding with the rise of philanthro-capitalism that is redefining the mandate of global institutions like WHO and World Trade Organization. The focus is now on finding ‘technical solutions’ to public health problems. This is well illustrated by the emergence of BMGF in the health sector and its alliances with global private capital.

The third theme that this paper highlights is how neoliberalism partners with religious fundamentalisms. The rise of religious fundamentalisms is a response to the social anxieties and inequalities that consumerism produces. Religious fundamentalisms impact gender differentially. The State becomes the key player in subjugating women with the reassertion of patriarchal values. Women’s bodies become the primary site for social control, which has negative consequences for reproductive choices as well as for sexual and reproductive health and rights. The alliance between neoliberalism and conservative politics and religious fundamentalisms has resulted in the control of women’s bodies and contributed to the reversal of hard-won rights for health and gender justice in many parts of the world.

## Abbreviations

BMGF: Bill and Melinda Gates Foundation
IMF: International Monetary Fund
NGO: non-governmental organisation
SAP: Structural Adjustment Programmes

## Footnotes

1 In the analysis of the engagement of the Structural Adjustment Programmes with developing countries, the impositional perspective argues that the conditionalities were forced by the World Bank and International Monetary Fund. This does not give agency to individual nation states in negotiating and contesting these conditionalities with the World Bank

2 This is based on the proceedings of the meeting of which Baru was a member of a Technical Advisory Group of the Ministry for Lymphatic Filariasis. The World Bank and WHO were pushing for the addition of albendazole to the existing regimen. The scientists on this committee opposed this move by citing relevant studies. It generated a great deal of debate among the scientists engaged with lymphatic filariasis.

3 In several countries in East Africa, the political class accepted the conditionalities with little negotiation with the World Bank and rapidly privatised their health services. Zambia is a case in point. For more details see Nanda [6].

4 This is based on debates between those for, against and sceptics of liberalisation during the 1990s. This was well represented in academic writings and debates in the print and visual media.

5 The Peoples Health Movement and its Indian chapter played an important role in resisting health sector reforms and also highlighting inequities. The Indian chapter of the Jan Swasthya Abhiyan has also played a role in highlighting inequities.

6 For example, the Peoples’ Health Assemblies organised by the global and national chapters of the Peoples Health Movement
